# Therapeutic Potential of Intravenous Ketamine in Early-Onset Dementia: A Case Report

**DOI:** 10.7759/cureus.65261

**Published:** 2024-07-24

**Authors:** Mariam Tadros, Dianella Rente Lavastida, Ashraf Hanna

**Affiliations:** 1 College of Medicine, Lake Erie College of Osteopathic Medicine, Bradenton, USA; 2 Infectious Diseases, St. George's University School of Medicine, True Blue, GRD; 3 Pain Management, Florida Spine Institute, Clearwater, USA

**Keywords:** anti-inflammatory, treatment-resistant, alzheimer's disease, neurology, early-onset dementia, intravenous, iv ketamine, dementia

## Abstract

This case report discusses the use of intravenous (IV) ketamine as a potential therapeutic agent for early-onset dementia. A 56-year-old female with a diagnosis of early-onset dementia showed significant cognitive decline despite trying and failing several standard treatments such as memantine, donepezil, and rivastigmine. Given the promising results of ketamine in other neurological and psychiatric disorders, the patient underwent a series of IV ketamine infusions over a period of two months. Following treatment, there was a notable improvement in cognitive function, mood, and daily living activities. By the end of her treatments, the patient stated she had more mental clarity, increased focus, improved memory, and increased energy. This case highlights the potential use of ketamine as a novel treatment approach for early-onset dementia and warrants further investigation in larger clinical trials.

## Introduction

Dementia is defined as a loss of cognition in two or more neurocognitive domains, such as executive function, complex attention, language, learning, memory, perceptual-motor, and social cognition. This leads to a loss of both social and occupational functioning and typically requires assistance in everyday activities [1]. It most commonly presents in people older than 68 years, although this can vary depending on the neuropathology. Diagnosing dementia requires a combination of cognitive assessments and collateral information from family members, yielding impairments in memory, attention, language, executive function, mood, and overall personality [1]. Physicians typically order and review MRI and CT scan results before diagnosing dementia to exclude other potential causes of memory changes, like bleeding or fluid accumulation in the brain [2]. Additional assessments, such as the CNS Vital Signs Neurocognitive Test, which measures the severity of impairment, can be used as a non-invasive procedure that aims to assess a broad range of neurocognitive domains under certain challenges. Utilizing tests and conducting regular neurocognitive assessments offers valuable insights for patients and their families, aiding in addressing challenges related to daily activities, educational pursuits, and professional endeavors [3].

Current management of dementia is individualized to the patient and mainly includes a combination of family support and medications that target symptoms. The most common agents used include acetylcholinesterase inhibitors such as galantamine, rivastigmine, and donepezil, which work by increasing the availability of acetylcholine neurotransmitters at the synapse [4]. For moderate-to-severe Alzheimer’s, the most common symptomatic treatment is memantine, a low-affinity N-methyl-D-aspartate (NMDA) receptor antagonist that inhibits calcium influx caused by overstimulation of the NMDA receptor, causing desensitization leading to a decrease in glutamate neurotoxicity [5]. Memantine’s mechanism of action is shared with ketamine, which acts as an NMDA-receptor antagonist and also operates across various stages of inflammation, influencing the recruitment of inflammatory cells, the production of cytokines, and the regulation of inflammatory mediators [6]. 

Ketamine is a well-known analgesic that was first introduced in the 1960s. Today, ketamine infusions are used and continue to be studied as a treatment option for conditions such as complex regional pain syndrome (CRPS), phantom limb pain, fibromyalgia, oncologic-induced neuropathic pain, and even refractory depression [7]. Studies have shown that ketamine can target behavioral disturbances such as agitation, catatonia, depression, and dementia [8]. This may be related to its non-competitive N-methyl-D-aspartate (NMDA) receptor antagonism, similar to memantine. Alzheimer’s disease (AD), the most common cause of dementia, is characterized by an accumulation of beta-amyloid in the brain that stimulates an inflammatory response, causing neuronal damage [8]. Considering this, utilizing ketamine as an anti-inflammatory regulator could potentially decelerate the advancement of Alzheimer’s disease.

Memantine is typically well-tolerated by Alzheimer’s patients but can cause symptoms of fatigue, gastrointestinal upset, and psychomotor agitation [4]. A recent study demonstrated that memantine preferentially inhibits NMDA receptors under neurotoxic conditions, whereas, in contrast, ketamine inhibits these receptors strongly in all contexts and is not restricted to only neurotoxic conditions [9]. Ketamine has been reported to cause dizziness, poor coordination, blurred vision, and visual or auditory hallucinations. However, all side effects of ketamine have been shown to resolve within four hours after administration, and there has not been evidence of any long-term medical effects thus far [10]. The short-term adverse effects reported with ketamine infusions may hinder further investigation of the drug as a therapeutic agent, though it has been proven that its procognitive and antidepressant effects are impactful. Whereas memantine’s side effects are less significant, the cognitive and behavioral effects are minimal [11]. 

Dementia imposes a profound burden on individuals and their loved ones, reshaping lives in a multitude of challenging ways. As cognitive functions decline, patients face a loss of independence, disruption in interpersonal relationships, financial strains, and overall emotional toll on patients and caregivers. A board-certified anesthesiologist and pain management doctor from the Florida Spine Institute has found that intravenous (IV) ketamine and its anti-inflammatory effects may provide beneficial results in patients with different neurocognitive and autoimmune conditions and decided to initiate treatment on the patient described in this case report.

## Case presentation

This case report presents a 56-year-old female who had a past medical history of bipolar/manic depression, anxiety, chronic pain disorder associated with rheumatoid arthritis, fibromyalgia, dementia, cystic fibrosis, and pancreatic insufficiency. Her husband first noticed her having memory impairment in 2020, stating that she was forgetful of recent events, could not recall recent conversations, asked the same questions repeatedly despite getting adequate answers, had difficulty with word finding, got lost in familiar surroundings while driving, and had instances of forgetting midsentence what she was saying. As time continued, they both noticed a loss of attention, increased confusion, brain fog, and personality changes. Subsequently, the patient's neurologist ordered a CT scan of the brain with and without contrast that showed no intracranial hemorrhage, transcortical infarction, mass, midline shift, hydrocephalus, or extra-axial fluid collection. There was no evidence of abnormal brain-enhancing lesions, and no acute intracranial abnormalities were found. This imaging was done to show the lack of acute reasoning for memory loss and to rule out any infarction or hydrocephalus that could be causing the patient's problems at that time. In August of 2022, she visited her neurologist again, who performed cognitive testing exams through the CNS Vital Signs Neurocognitive Test that revealed a very low for-age neurocognitive index exemplified by difficulty with attention span, reaction time, psychomotor speed, and multitasking. She scored in the first percentile (very low score) in neurocognitive index, 16th percentile (low average score) in composite memory, 10th percentile (low average score) in visual memory, first percentile (very low score) in executive function, and fourth percentile (low score) in reaction time. The neurologist concluded that she had early-onset dementia without behavioral disturbances, likely due to early-onset Alzheimer's disease. She was subsequently started on memantine 10 mg, and shortly after, stated that it made her more confused and increased her brain fog. Therefore, it was decided to discontinue this drug and start donepezil 5mg, which she also did not tolerate due to nausea, fatigue, and depressed mood. Finally, she was put on rivastigmine 1.5 mg, another type of acetylcholinesterase inhibitor, which she generally tolerated but complained of fatigue [12]. With this treatment, neither she nor her husband saw any significant improvement in her cognitive status.

In September of 2022, she began seeing a pain management specialist at the Florida Spine Institute for chronic pain due to fibromyalgia and rheumatoid arthritis that had been worsening despite her treatment regimen at that time. During this visit, the doctor first suggested ketamine treatment for her pain as well as her depression. In October, she began taking 30 mg ketamine capsules daily, which was then increased to three times daily in December. On December 19, 2022, she began a 10-day IV ketamine infusion per protocol as a treatment for her depression. She reported that this was her first time ever trying ketamine treatment and immediately saw hopeful results. These infusions were administered according to the pain management doctor's prescribed protocol, using a starting dose of 150 mg of ketamine alongside 10 mg of lidocaine, 4 mg of ondansetron, and 25 mg of promethazine to prevent nausea and vomiting. She successfully completed this treatment with no adverse effects and reported improved mood and energy by the end of the first round of infusion. By her third treatment, she reported great improvement in her depression, resulting in increased socialization and activity compared to her usual state. In January of 2023, she continued using ketamine hydrochloride (HCL) powder and was prescribed 30 mg ketamine lozenges three times daily. She continued follow-up infusions for two days per week.

In August of 2023, the idea of using IV ketamine for this patient’s early-onset dementia was thought of by the pain management specialist at Florida Spine Institute and was discussed with the patient and her husband. After being successful with her depression, the aim now was to slow the progression of her dementia and clear her brain fog. They agreed to try this treatment and went back to her independent neurologist for cognitive testing. On August 7, 2023, the patient received in-depth cognitive testing that showed a low average neurocognitive index. She had the most difficulty with reaction time, psychomotor speed, visual memory, and composite memory. This appointment served as the baseline cognitive function testing before restarting the IV ketamine infusions. In September of 2023, she began receiving three infusions per week every two weeks, as well as taking ketamine capsules daily. After the first round of infusions, she reported decreased depression, decreased brain fog, and increased mental clarity. Later, she was able to decrease her dosage of quetiapine for bipolar disorder and depression from 400 mg twice daily to 400 mg once at bedtime. The rivastigmine prescribed for her dementia was also successfully discontinued due to her cognitive improvement.

**Figure 1:**
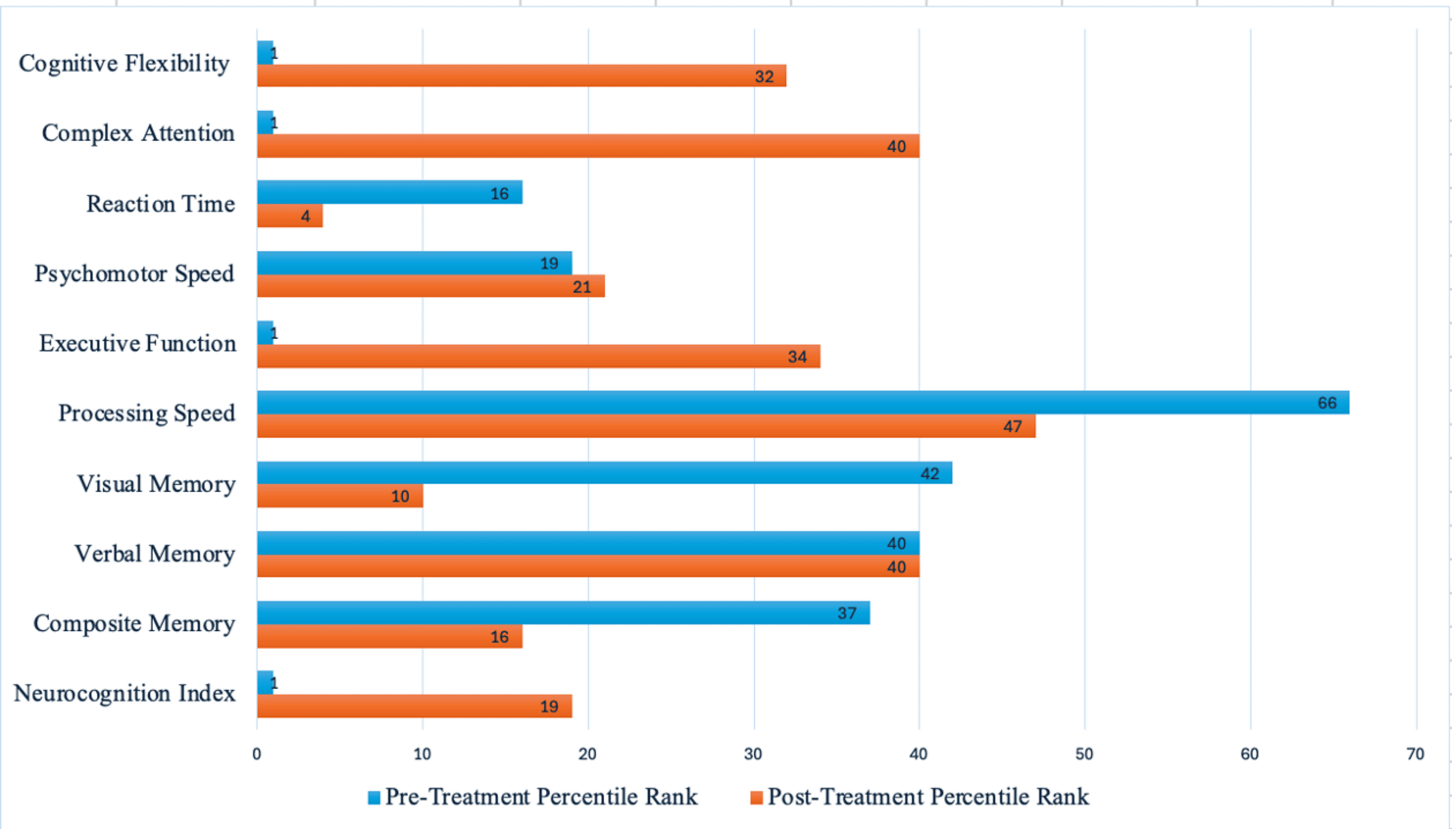
CNS Vital Signs Neurocognitive Testing Report pre-ketamine treatment and post-ketamine treatment. Above-average domain scores indicate a Percentile rank (PR) greater than 74, indicating a high-functioning test subject. The average is a PR of 25-74, indicating normal function. A low average is a PR of 9-24, indicating a slight deficit or impairment. Below average is a PR of 2-8, indicating a moderate level of deficit or impairment. Very low is a PR less than 2, indicating a deficit and impairment. The CNS Vital Signs Standard Scores and Percentile ranks are auto-scored using an algorithm based on a normative data set of 1600+ subjects, ranging from ages 8 to 90. In the age-matched normative sample subjects were: (1) in good health, (2) had no past or present psychiatric or neurological disorders, head injury, or learning disabilities, and (3) sample subjects were free of any centrally acting medications [3].

**Figure 2:**
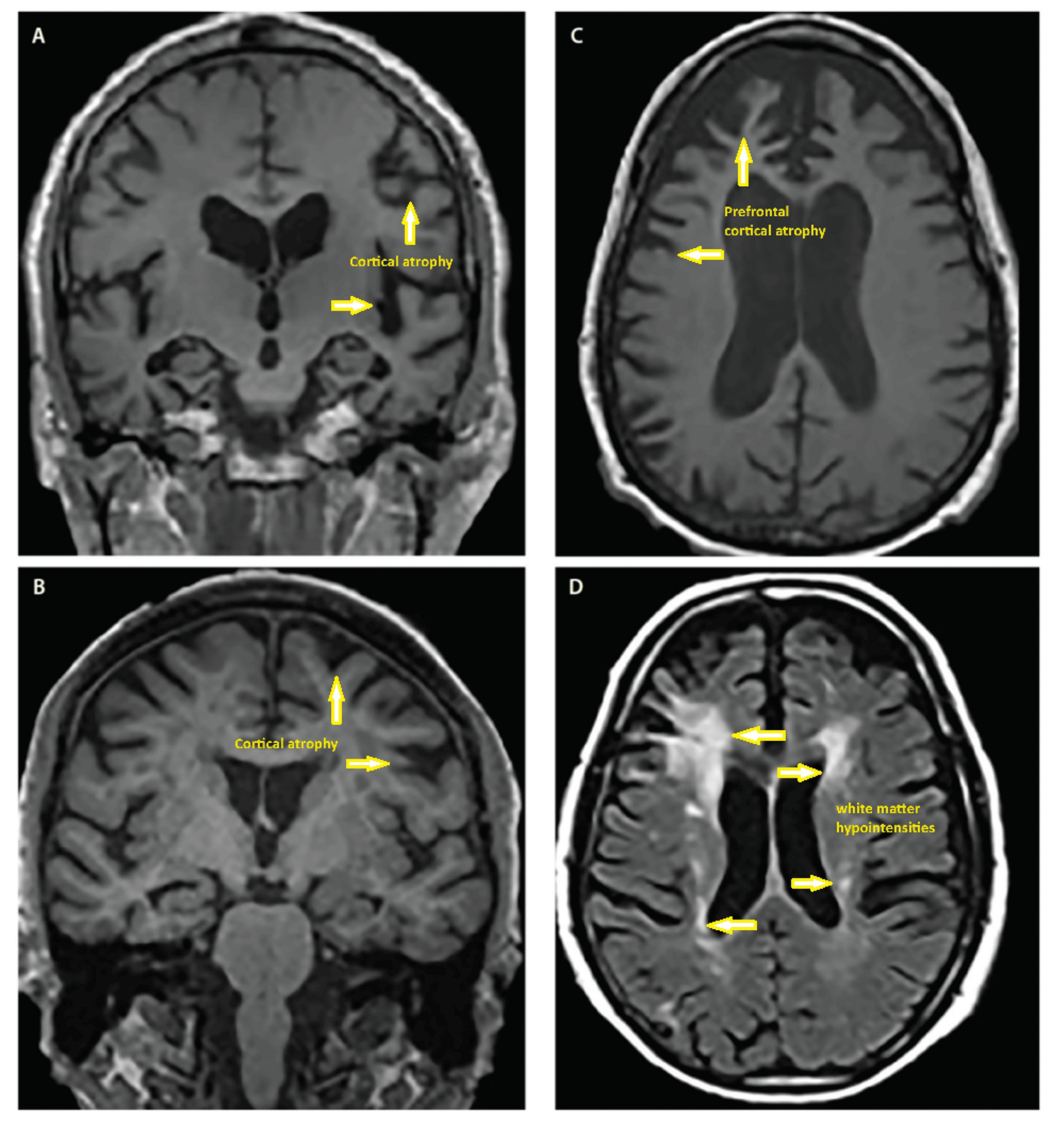
MRI images of dementia. Structural atrophy patterns across Alzheimer’s disease (AD) (A), dementia with Lewy bodies (DLB) (B), frontotemporal dementia (FTD) (C), and vascular dementia (VaD) (D). Coronal T1-weighted MRI demonstrates generalized cortical atrophy in AD (A) and DLB (B). Axial T1-weighted MRI demonstrates biventricular frontal and temporal atrophy with a predilection for the medial and lateral prefrontal cortices (C). Axial T2-weighted MRI demonstrates confluent periventricular cap and halo white matter hypointensities involving the corona radiata, lateral prefrontal neocortex, subependymal ventricular lining and septum pellucidum in VaD (D) [13].

On October 4th, 2023, the patient reported her memory improved greatly for one week, then gradually had her confusion return. After her third ketamine infusion of that week, she reported being able to think more effectively and clearly and was able to multitask in her daily activities. After just two months of treatment with IV ketamine infusions, another independent neurologist performed a Dementia Rating Scale (DRS) and found only mild cognitive impairment, likely due to focus impairment rather than memory impairment. Shortly after this, her ketamine capsule dosage was changed to 15 mg every eight hours, and she continued using the ketamine lozenges. Her infusions started at three days every two weeks, then progressed to two days every three weeks, finally leading to two days every four weeks for booster treatments. In November, the patient again reported that she felt more mental clarity and less brain fog, resulting in increased focus and improvement of cognitive function. On November 9th, her second cognitive test that was performed after beginning ketamine was reported by the neurologist who found significant improvement compared to the previous testing done in August of 2022, prior to beginning ketamine (Figure 1). Improvement was seen in cognitive testing in areas of neurocognitive index from the first percentile (very low score) to the 19th percentile (low average), composite memory from the 16th percentile (low average) to the 37th percentile (average), visual memory from the 10th percentile (low average) to the 42nd percentile (average), executive function from the first percentile (very low score) to the 34th percentile (average), and reaction time from fourth percentile (low score) to the 16th percentile (low average). The patient reported that she felt lighter, had better memory, experienced more energy, was able to multitask, felt significant mental clarity, and had been able to sleep well compared to her mental and physical state before beginning treatment. The patient continued to take 30 mg lozenges as needed and 15 mg ketamine capsules every eight hours, but eventually discontinued treatment because of the cost. After discontinuing, she states her previous state of fogginess and forgetfulness has slowly returned, and she is currently working to begin treatment again. Figure 2 demonstrates typical representations of different types of dementia seen on MRI, such as Alzheimer's disease (A), dementia with Lewy bodies (B), frontotemporal dementia (C), and vascular dementia (D) and the areas of neurodegeneration that would be seen in chronic stages of each disease [13]. Pertinent to our patient, because of the onset and timing of her impairment, CT and MRI may be negative and cannot be used to confirm or deny her disease. In later stages of Alzheimer's disease and dementia, MRI imaging could be expected to reflect specific areas of neurodegeneration, such as those in Figure 2. 

## Discussion

Dementia is one of the most challenging neuropsychiatric conditions to treat, given its degenerative nature, the comorbidities it presents with, and the lack of disease-modifying medications available. Ketamine, a non-conventional treatment, offers hope for this challenging disease. This report highlights a ground-breaking potential disease-modifying treatment for dementia, the first of its kind. 

This narrative reports the clinical course of a patient with presenile dementia due to suspected Alzheimer's disease who showed significant cognitive improvement after just two months of treatment with ketamine. Studies have shown the beneficial effect of ketamine on depression in Alzheimer’s patients. Evidence suggests there may be an overlap between depression and Alzheimer’s Disease because they share NMDA-regulated signaling pathways, hence the significant improvement of depression in Alzheimer’s patients [12]. Previous research has yielded an abundance of studies delineating the potential benefit of ketamine on depression, but not many on the impact of ketamine on the cognitive manifestations of dementia. Ketamine has been found to have a significant effect on inflammation by blocking cytokine production and inflammatory cell recruitment, thus leading to a profound and systemic anti-inflammatory response [6]. Due to the pathophysiology of AD stemming from an inflammatory response caused by amyloid deposition, using ketamine as an anti-inflammatory modulator may have led to the improvement and slowing of the disease in this patient. This case report is the first of its kind to show the beneficial usage of IV ketamine in the treatment of dementia and proves the need for further research and larger clinical trials on the impact ketamine may have on cognition and memory. 

Past studies have been done on the effects of ketamine related to psychomotor and neurological function, but not many have been done on its effect on neurocognitive degeneration. One study found positive effects on individuals with dementia with behavioral disturbances such as acute agitation, depression, and catatonia, with no significant adverse effects reported [14]. Other studies report a reduction of psychiatric distress in major depressive disorder (MDD), post-traumatic stress disorder (PTSD), bipolar disorder, obsessive-compulsive disorder (OCD), eating disorders, and delirium prevention but without a full understanding of the mechanism of action behind the effects of ketamine on cognitive function [15,16]. Cognitive testing on individuals suffering from MDD and PTSD demonstrated improvement in psychomotor function, visual attention, visual learning, visual localization, acquisition, and overall memory [16]. 

Some factors that hinder further study with ketamine include the potential short-term side effects, the stigma surrounding the drug, and the cost of administration. Dizziness, poor coordination, blurred vision, and brain fog are short-term adverse effects that have been reported by those who have trialed ketamine for depression [8,10]. The classification of ketamine as a Schedule III controlled substance also raises concerns for the safety of usage [17]. Furthermore, this treatment is generally not covered by insurance, requiring many patients to pay out of pocket and resulting in a significant financial burden [17]. In this case, the patient had to discontinue her treatment because of the cost, and she was no longer able to afford it. Some studies performed on mice suggest that ketamine may even lead to the onset of Alzheimer’s Dementia. One study found ketamine to potentially induce phosphorylation of tau protein in the hippocampus of mice, leading to cognitive deficits [18]. These aforementioned factors are important barriers in the treatment with IV ketamine. Further trials and case reports are essential to emphasize the beneficial effects of ketamine, paving the way for adjustments to mitigate these barriers and enable broader utilization of IV ketamine in addressing neurocognitive, psychiatric, and autoimmune disorders.

## Conclusions

This case report demonstrates the potential of ketamine to improve symptoms of early-onset dementia, as evidenced by MRI findings. Notably, the patient had previously been treated with ketamine for treatment-resistant depression, which may have influenced the observed improvements in her dementia symptoms. Further research to understand the true mechanism by which ketamine has an impact on neurologic function, such as its anti-inflammatory effect, is imperative for the inclusion of ketamine in the treatment of these diseases. With increasing research, ketamine can be widely incorporated for the treatment of conditions such as early-onset dementia and Alzheimer’s disease, conditions in which commonly used medications have minimal impact. We encourage colleagues to implement trials with IV ketamine to treat neurodegenerative and neuropsychiatric conditions to add to the literature in support of ketamine as a potential long-term solution. 
